# Needs Assessment Lessons Learned in Qatar: A Flipped Classroom Approach

**DOI:** 10.15694/mep.2019.000048.1

**Published:** 2019-03-12

**Authors:** Gabriela Berger

**Affiliations:** 1 Sidra Medicine

**Keywords:** Continuing Health Professions Education, Flipped Classroom, Needs Assessment, Adult Learning

## Abstract

This article was migrated. The article was marked as recommended.

Continuing professional development (CPD) activities are expected to be purposeful and effective; the essential first step is a curriculum based on actual training needs. The Qatar Council for Healthcare Practitioners (QCHP) provides the oversight for accredited CPD programs for all groups of healthcare practitioners in Qatar this includes physicians, pharmacists, nurses and allied health professionals. In December 2017 several professionals created a local, collaborative and supportive network to share their expertise and to collaborate on accredited professional development initiatives. Network members included accredited CPD providers from two hospitals, Sidra Medicine and Aspetar, and from the College of the North Atlantic Qatar (CNA-Q). The implementation of a needs analysis is key to continuous service improvements. Network members conducted an audit of 43 educational programs in 2018 and found that this step is frequently missed or misinterpreted by curriculum planners. A workshop on needs assessments was seen as the most germane intervention to upskill program planners. An evidence-based approach was suggested in the development of a needs assessment teaching module. A review of the literature influenced the curriculum design as did a re-examination of adult learning principles and validated new methods to teaching in the health professions. The flipped classroom was identified as the most appropriate approach regarding design and content delivery. It requires skilled facilitators and content matter experts that are familiar with diverse teaching formats to ensure that learning outcomes are integrated across the formats. It has been documented that ‘flipping’ the classroom from a teacher-centered to a learner-centered approach encourages participants’ higher order learning and fosters engagement, exploration, evaluation and reflection.

## What problems were addressed?

Due to dynamic changes of health care needs the health workforce requires continuous training of relevant skills. Evidence-based approaches are the cornerstone of all training programs and this includes the development, and application of needs analysis tool to determine training needs. This is an often missed essential first step in instructional design.

There are limited opportunities for curriculum development training in Qatar. In December 2014 Weill Cornell Medicine-Qatar convened a two-day event for 200 healthcare professionals that presented renowned author and Emeritus Professor Dr John Kern’s (Johns Hopkins University School of Medicine) Six-Step Curriculum Model ( Cantillon et al., 2017). There is opportunity to offer comprehensive training workshops that address various aspects of curriculum development such as needs assessments, the formulation of learning objectives and research design. The combination of practitioner training needs, lack of local training opportunities and the benefits of collaboration provided the impetus for creating a supportive network.

During the 2018 calendar year, needs assessments from three CPD providers in Qatar, Sidra Medicine, Aspetar and CNA-Q, were analyzed. It showed that the definition of ‘needs assessment’ varied and was mostly interpreted in terms of ‘what staff are interested in and want to know more about’. An analysis of 43 needs assessments prepared by educational planners showed considerable variation in the types of questions asked, the description and the analysis of findings. A systematic approach to the reporting of research findings was absent. ‘Interest’ in educational topics and knowledge ‘gaps’ were used synonymously. There was patchy understanding of how to conduct a needs analysis, the types of questions to ask, and methods required to elicit a lack of knowledge and practice gaps. Surveys were the primary tool used for data collection. It was not clear to the planners why various practitioner groups that were part of the target audience needed to participate in the needs assessment. The idea of educational planning was largely resisted because in the absence of a needs assessment, it was not possible to identify learning topics from identified gaps, and thus the curriculum could not be designed in advance of a CPD initiative.

A sound needs analysis provides the foundation for development of educational activities. It shapes the curriculum, enhances in-service training and ultimately aims at providing excellence in patient care. The health workforce in Qatar is culturally diverse, with a significant turnover rate, and includes practitioners from various streams such as medical, nursing, pharmacy and allied health who work in the public and private healthcare sectors. Practitioners have undergone training world-wide to various competency levels in clinical research and teaching. For example, at Sidra Medicine, health professionals originate from over 90 countries, have completed their training in a variety of educational institutions on various continents, and range from novice to (world) expert in a given health professional field that ranges widely from functional genomics research to nursing and occupational health. The majority of educational planners were not trained in research methodology and therefore needed guidance and support in applying needs analysis.

Adult learners require active participation in a learning activity, they are able to diagnose their own learning needs and thus can formulate their learning, they are self-directed and highly motivated, but require triggers for reflection on action ( Kaufman, 2003). In the teaching context this means that adults are independent, have accumulated considerable experience, are interested in problem-solving approaches rather than subject-centered approaches and have high levels of internal and external motivation.

Teaching needs assessments one-on-one is time-consuming and lacks the positive influence of peer interaction. A group learning approach with high quality developed resources would be beneficial due to the positive influence of peer teaching and the presence of trained educators to facilitate the process. The needs assessment training module was thus divided in specific problem-based sections that gave learners the opportunity to hear about subject content and the opportunity to apply the learning theory to real scenarios. Peer learning is encouraged and active participation is fostered, with no more than 20 minutes per hour spent on discussing the subject and the remainder on learning how to design various elements of the needs assessment process.

## What was tried?

For this study we developed a needs analysis training model that is congruent with current educational approaches in health professional education. The teaching pedagogy of the flipped classroom has become increasingly popular as it supports preferred adult learning approaches of problem-based learning (PBL) and case-based learning (CBL). ‘Flipping’ the classroom means to move away from teacher-centered to learner-centered strategies: subject information is provided as pre-reading materials that allow learners to become more engaged during in-class activities. ( Persky and McLaughlin, 2017, Swanwick and Association for the Study of Medical, 2013). The off-loaded content is part of the pre-reading package and in this digital age, information can readily be accessed through video lectures, podcasts and traditional readings or handouts. This blended teaching approach appeals to different types of learners, promotes self-paced learning, and allows for peer and instructor engagement. A meta-analysis of 28 comparative studies has found that learners prefer the flipped classroom to traditional approaches, and show significant improvements in learning outcomes. In addition, quizzes at the start of each in-class sessions were reported to be particularly effective ( Hew and Lo, 2018).

The flipped classroom requires an evidence-based approach in regard to the instructional module, with a clear definition of learning objectives for all learning activities so that learners can achieve the desired outcomes; this represented a challenge. Over several months we collaborated on a workshop design that showed clear structure with sequenced units by increasing difficulty towards mastery. Transfer from theory to practice was achieved by engagement in practical activities for each teaching segment. At the beginning of each teaching segment (three segments over three hours) a pre-quiz was prepared that monitors pre-teaching learning experiences including errors and levels of understanding; this affords deliberate practice for further advancement.

## What lessons were learned?

We designed and co-facilitated the needs assessment workshops. These are ongoing and available to all CPD developers in Qatar. The most challenging aspect of teamwork is not so much the geographical dispersion in Qatar but the fact that we were each trained on a different continent and educational institution, and we hold different expertise and jargons ( Edmondson, 2012). We crossed cultures, priorities and values; this we incidentally require from our participants. We needed time to build trust and mutual understanding, with constant communication and coordination. We also needed to feel safe to speak up honestly by asking questions, raising concerns, and explaining ideas. This experimental approach required us to listen to each other, to synthesize different points of view to create new possibilities. Moments of reflection provided new opportunities for learning and change, and the challenges will bring rewards. We will be able to develop a broader knowledge of the subject area, we may attract a bigger network of potential collaborators, the future needs assessment workshop facilitators, and we will have developed our own ‘knowledge marketplace’ with resources that we can share to promote continuous medical education.

## Take Home Messages

• Adult learners respond well to self-paced, blended learning modules
• The flipped classroom is increasingly used in health professions teaching
• Pre-and post-session readings and reflective evaluations boost memory retention
• Interactivity is of primary importance and allows peer teaching on needs assessments
• This education is created by the healthcare team for the team for continued learning and change

## Notes On Contributors

Dr Gabriela Berger is a medical anthropologist with a PhD from Griffith University, in Brisbane, Australia; she published her thesis under the title of *Women and Menopause* (Pluto Press, 1999). She has published widely in peer reviewed journals and her research interests include medical education and accrediation, continuing professional development, cross-cultrual studies, cultural competence, health literacy, women’s reproductive health, health promotion and the Mediterranean diet. She currently oversees the continuing professional development program at Sidra Medicine in Doha, Qatar.

Author ORCID Number: https://orcid.org/0000-0002-8706-5231
