# Supplementary material for: Needs Assessment Lessons Learned in Qatar: A Flipped Classroom Approach
Source: MedEdPublish (2016). 2019 Mar 12;8:48. [Version 1] doi: 10.15694/mep.2019.000048.1 (PMC10712580; doi:10.15694/mep.2019.000048.1)
Supplement: Peer review history

# Reviewer report: Reviewer response for version 1

**DOI:** 10.21956/mep.19787.r29745

**Published:** 2019-07-03

**Authors:** Ken Masters

**Affiliations:** 1 Sultan Qaboos University

This review has been migrated. The reviewer awarded 3 stars out of 5 A short and interesting report on a needs-assessment workshop from Qatar. The report explains succinctly how needs assessments from CPD providers were used as a basis for the workshop, the workshop was designed and various lessons learned. For the report to have greater value, however, it would have been useful if the author had supplied more detail. The design, running and achievements are described in very board outline only, and it would be valuable if the author had given some more detail on the elements extracted from the providers, the curriculum/schedule of the workshop, and some form of evaluation results.So, the report is tantalising, but really does need to give some more detail in order for readers to have insight into its strength and value.

Reviewer Expertise:

NA

No decision status is available

# Reviewer report: Reviewer response for version 1

**DOI:** 10.21956/mep.19787.r29744

**Published:** 2019-03-30

**Authors:** Helena Filipe

**Affiliations:** 1 Hospital of the Armed Forces/PL-EMGFA

This review has been migrated. The reviewer awarded 4 stars out of 5 The manuscript focuses on good practice in continuing professional education (CPD), namely in creating effective CPD programs. Identified the need of developing skills in needs assessment as a missing step by curriculum planners of a network of three organizations in Qatar and analyzed the gap to close a workshop in needs assessment skills was designed. The flipped classroom was the educational strategy chosen.The Author elaborates on what a good needs assessment entails, on the theoretical concept beneath the flipped classroom and how it applies in the present case to conclude with the process set in place. Best acknowledgement to the Author for sharing such insight and evidence informed planning experience. It will be interesting to see in the future a paper on results of this ongoing program.

Reviewer Expertise:

NA

No decision status is available
